# Identification of RAGE and OSM as New Prognosis Biomarkers of Severe Pneumonia

**DOI:** 10.1155/2022/3854191

**Published:** 2022-01-07

**Authors:** Jing Lei, Li Wang, Qian Li, Lin Gao, Jing Zhang, Yan Tan

**Affiliations:** Department of Respiratory and Critical Medicine, Nanjing First Hospital, Nanjing Medical University, Nanjing 210000, China

## Abstract

**Objective:**

To investigate efficiency of RAGE and OSM as new prognosis biomarkers of severe pneumonia.

**Methods:**

Eligible patients were classified into hypoxemia and nonhypoxemia groups. Meanwhile, the same cohort was divided into survival and nonsurvival groups after a post-hospital stay of 30 days. We analyzed risk factors for the hypoxia and death among these patients.

**Results:**

Compared with nonsurvival group, significant increase was noticed in PH, lymphocyte, albumin and platelet level in survival group, while significant decline was noticed in neutrophils, RBC, hemoglobin, hematocrit, creatinine, total bilirubin, CRP, PCT, OSM, RAGE and neutrophils/lymphocyte level. Oxygenation index level was related to APACHE II, LIS, SOFA, NUTRIC score, WBC, neutrophils, lymphocyte, RAGE, and albumin level (*p* < 0.05). LIS, SOFA, NUTRIC score, lac, lymphocyte, platelet, BUN, total bilirubin, PCT, and OSM levels were associated with mortality rate (*p* < 0.05).

**Conclusions:**

RAGE and OSM may serve as a new biomarker for poor prognosis in pneumonia patients.

## 1. Introduction

Pneumonia refers to a common disease that is usually associated with a poor treatment outcome. Patients with severe pneumonia usually show hypoxic respiratory failure, leading to a high morbidity and mortality. In the past decades, extensive efforts have been made on the diagnosis and treatment of pneumonia, in order to improve the outcome among these cases.

Increasing evidence indicates that laboratory biomarkers can be used for diagnosis and outcome prediction in infectious diseases and a variety of medical conditions including major cardiac events, cerebral hemorrhage, and cancer. Tamhane et al. proved that the neutrophil/lymphocyte ratio (NLR) was an independent predictor for mortality in patients who underwent percutaneous coronary intervention [1]. In addition, Lattanzi et al. [2] found that the NLR was associated with 30-day mortality and morbidity of patient with acute intracerebral hemorrhage, and improved the accuracy of outcome prediction. Howard et al. proved that NLR was associated with poorer survival outcomes in patients with solid tumor [3]. Moreover, patients with ischemic stroke who underwent endovascular treatment (EVT) showed higher systemic inflammatory response index (SIRI) at admission, and were at increased risk of poor outcome [4].

Patients with severe pneumonia usually show concomitant diseases such as cerebrovascular disease (CVD), diabetes mellitus (DM), coronary heart disease (CHD), chronic renal damage, and nasopharyngeal carcinoma. Indeed, the concomitant diseases closely associated with hypoxia have been acknowledged as risk factors for the progression of pneumonia. To date, there is still a lack of specific and sensitive markers for early identification of patients with increased risks of severe pneumonia, especially those complicated with hypoxic respiratory failure.

The receptor for advanced glycation end products (RAGE), a 35 kDa protein from the immunoglobulin superfamily that propagates the inflammatory response via NF-*κ*B [5], is a marker of type I alveolar epithelial cell injury in rats and patients with acute respiratory distress syndrome (ARDS) [6]. In a previous study, plasma RAGE level showed decline in lung-protective ventilation in patients undergoing major abdominal surgery compared to those who received nonprotective ventilation [7]. Meanwhile, a high plasma RAGE was associated with an increased mortality in patients with acute lung injury [8]. Unfortunately, little is known about the roles of RAGE in the pathogenesis and prognosis of pneumonia. Oncostatin *M* (OSM) receptors are a member of the IL-6 family, involving in endothelial damages that contribute to increased permeability to liquid and protein and consequent edema in interstitium space [9]. Neutrophil-released OSM affected endothelial cellular function under both physiological and pathological conditions [10]. Despite its significance in pathogenesis of certain diseases, the feasibility of OSM as a biomarker for predicting the disease severity and prognosis is still not well defined. In this study, we investigated the feasibility of RAGE and OSM as new biomarkers for predicting the outcomes in patients with pneumonia.

## 2. Materials and Methods

### 2.1. Participants

This study was performed in the respiratory intensive care unit (RICU) of our hospital. All clinical data were retrospectively reviewed for pneumonia patients admitted to the RICU from January 2017 to October 2019. Patients included in our study presented at least one acute symptom (e.g. breathlessness, cough or fever). Besides, they showed pulmonary infiltration based on chest high resolution computed tomography. Patients with other lung complications such as *tuberculosis*, acute pulmonary embolism, congenital heart disease, and untreated aggressive carcinoma were excluded from this study.

### 2.2. Study Design

Patients were divided into two groups based on oxygenation index: group 1 with oxygenation index of >250 mmHg and group 2 with oxygenation index of ≤250 mmHg. After RICU admission, patient characteristics including disease course, medical history, smoking, drinking alcohol, physical and vital signs, hospital stay, prognosis, and medication histories were collected on days 1 and 7, respectively. Besides, the treatment options (e.g., oxygen usage and MV setting) were also collected. Furthermore, laboratory indices were measured, including blood cell count, coagulation analysis, CRP, PCT, OSM, RAGE, and T cell subtypes, as well as hepatic, renal, and cardiac tests.

We measured the lung injury score (LIS), NUTRIC score, gas exchange, as well as organ failure. Specifically, LIS was calculated based on oxygenation index, the area of pulmonary infiltration on chest X-ray, positive end expiratory pressure (PEEP), and pulmonary compliance. NUTRIC score was calculated based on age, APACHE II score, sequential organ failure assessment (SOFA), concomitant diseases, time from admission to RICU, and IL-6.

According to the prognosis, patients were separated into survival and nonsurvival groups. Patients with pneumonia that had different outcomes were compared in terms of clinical, laboratory and prognostic characteristics on day 1 and day 7 after admission. We then analyzed the related risk factors for the mortality.

### 2.3. Statistical Analysis

Statistical analysis was performed using SPSS 22.0 software. Continuous variables were presented as mean ± standard deviation, and categorical variables were summarized as frequency and percentages. Independent-sample Student's *t*-test was utilized for intergroup comparison of continuous variables, while Chi-square test or Mann–Whitney test was used for intergroup comparison of categorical variables. Single factor Logistic regression analysis was conducted for the identification of risk factors. The area under receiver operating characteristic (ROC) curves indicated a strong predictive power for the biomarkers' area, which represented the largest area under the curve (AUC). Pearson product-moment correlation analysis was given to investigate the relationship between these markers and the survival or severity of pneumonia. *p* < 0.05 was considered to be statistically significant.

## 3. Results

### 3.1. Patient Characteristics

Among the 828 cases admitted into the RICU of our hospital, 130 patients (15.70%) were enrolled in our final analysis and were divided into group 1 with oxygenation index of >250 mmHg (*n* = 76) and group 2 with oxygenation index of ≤250 mmHg (*n* = 54). There were no statistical differences in the baseline information of these patients in both groups. Among the patients, 16 were dead, and 112 survived.

### 3.2. Comparison of Gender and Concomitant Diseases, Age and Clinical Scores, and Clinical Scores

The proportion of cases with DM in the group with oxygenation index of ≤250 mmHg was significantly higher compared with the group with oxygenation index of >250 mmHg. In addition, the male was more likely to develop hypoxemia (Table 1). For the concomitant diseases, there were no statistical differences in the proportion of patients with CVD, CHD, chronic renal damage, and nasopharyngeal carcinoma between the two groups (*p* > 0.05, Table 1).

There were no statistical differences in age between two groups. APACHE II, LIS, SOFA and NUTRIC scores showed significant increase in the patients with an oxygenation index of ≤250 mmHg compared to those with an oxygenation index of >250 mmHg (*p* < 0.05, Table 2). Compared with patients with an oxygenation index of >250 mmHg, there was significant increase in the white blood cell (WBC) count, neutrophils, neutrophils/lymphocyte ratio, lactic acid, creatinine, D-dimer, PCT, CRP and RAGE levels in patients with an oxygenation index of ≤250 mmHg (*p* < 0.05, Table 3). In contrast, the PH, lymphocyte and albumin levels were significantly lower in the patients with an oxygenation index of >250 mmHg compared with the counterparts with an oxygenation index of ≤250 mmHg (*p* < 0.05). There were no significant differences in PaCO_2_, hematocrit, hemoglobin, platelet, erythrocyte, blood urea nitrogen (BUN), lactate dehydrogenase (LDH), total bilirubin, prothrombin time (PT), activated partial thromboplastin time (APTT), fibrinogen (Fib) and OSM level between two groups.

### 3.3. Comparison of Parameters between the Survival and Nonsurvival Groups

Table 4 showed the comparison of gender and concomitant diseases between the survival and nonsurvival groups. There were no statistical differences in the gender, CVD, DM, CHD, chronic renal damage, and nasopharyngeal carcinoma between two groups (*p* > 0.05). For the comparison of age and clinical scores between the two groups, there was no statistical difference in age and APACHE II score between two groups (*p* > 0.05). In contrast, the LIS, SOFA, and NUTRIC scores were significantly higher in the nonsurvival group compared to those of the survival group (*p* < 0.05, Table 5). Compared to the survival group, significant increase was seen in the levels of lactic acid (*p*=0.008), BUN (*p*=0.002), total bilirubin (*p*=0.009), PCT (*p*=0.012), and OSM (*p*=0.001) in the nonsurvival group (Table 6).

### 3.4. Comparison of Laboratory Findings in Survival or Nonsurvival Groups before and after Treatment

Tables 7 and 8 summarize the changes of laboratory findings before and after treatment in the survival and non-survival groups, respectively. Compared with the baseline levels, the PH, lymphocyte, albumin, and platelet levels showed significant increase after treatment in the survival group (*p* < 0.05), while the number of neutrophils, red blood cells (RBCs), hemoglobin, hematocrit, creatinine, total bilirubin, CRP, PCT, OSM, RAGE, and neutrophils/lymphocyte ratio showed significant decrease compared with the baseline levels (*p* < 0.05).

It is important to point out that even though CRP and PaCO_2_ raised similarly to the survival group, the PH went lower in the nonsurvival group after treatment (*p* < 0.05). Meanwhile, Lac, WBC, neutrophils, lymphocyte, RBC, hemoglobin, hematocrit, platelet, albumin, creatinine, BUN, total bilirubin, PT, APTT, FiB, PCT, OSM, RAGE, and neutrophils/lymphocyte ratio showed no significant changes after treatment in the nonsurvival group.

### 3.5. Factors Correlated with Oxygenation Index and Survival

In this section, we determined the relationship between the oxygenation index and a serial of variables including scores of APACHE II, LIS, SOFA, NUTRIC, PH, lac acid, WBC, neutrophils, lymphocyte, neutrophils/lymphocyte, creatinine, RAGE, and albumin, respectively. Our data indicated that the oxygenation index was correlated with APACHE II, LIS, SOFA, NUTRIC scores, WBC, neutrophils, lymphocyte, RAGE, and albumin levels, respectively (*p* < 0.05). In addition, LIS, SOFA, NUTRIC scores, lactic acid, lymphocyte, platelet, BUN, total bilirubin, PCT, and OSM levels were correlated with the mortality (*p* < 0.05).

### 3.6. Correlation between Oxygenation Index and RAGE

**Figure 1:**
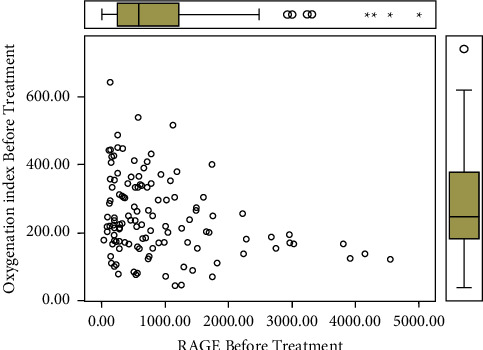
In order to examine the relationship between oxygenation index and the RAGE, we calculated the Pearson correlation coefficient, with −0.228, *p*=0.001 indicating a meaningful negative correlation between RAGE and oxygenation index.

To investigate the relationship between oxygenation index and RAGE, we calculated the Pearson correlation coefficient.  Figure 1 shows that oxygenation index was negatively correlated with RAGE (*r* = −0.228, *p*=0.001).

### 3.7. Correlation between Pneumonia Mortality with OSM

**Figure 2:**
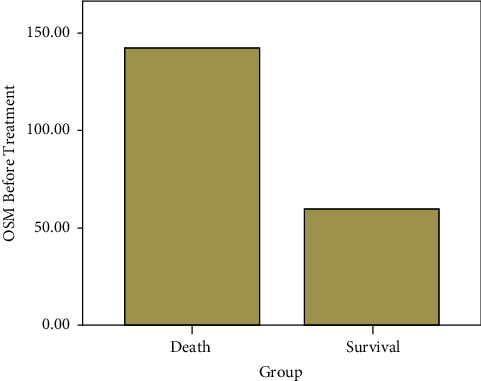
OSM level before treatment was associated with the pneumonia mortality. The Pearson correlation coefficient was −0.228, and *p*=0.001.

OSM level was correlated with the pneumonia mortality before treatment (*r* = −0.228, *p*=0.001Figure 2). At the baseline level, BUN, bilirubin, and platelet levels were correlated with survival. Nevertheless, CRP and PCT were correlated with survival after treatment.

**Figure 3:**
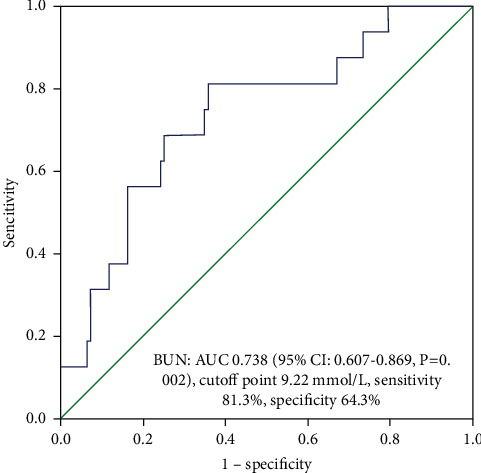
The AUC of BUN before treatment was calculated to be 0.738 (95% Cl: 0.607–0.869, *p*=0.002) with a cutoff point of 9.22 mmol/L yielding a sensitivity and specificity of 81.3% and 64.3%, respectively.

For the cutoff value of BUN prior to treatment, AUC of BUN was 0.738 (95% CI: 0.607–0.869, *p*=0.002) with a cutoff point of 9.22 mmol/L. This yielded a sensitivity and specificity of 81.3% and 64.3%, respectively (Figure 3).

**Figure 4:**
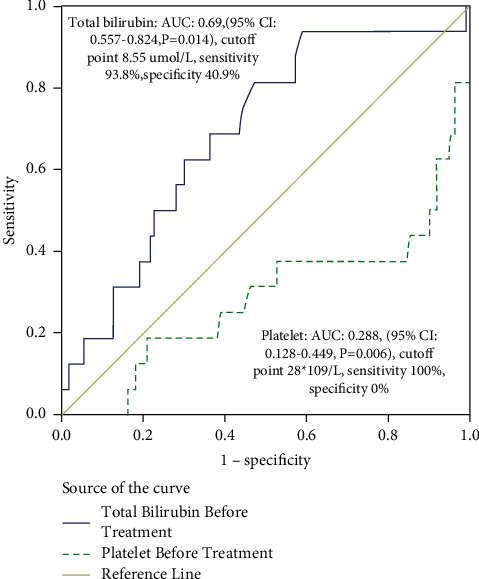
ROC curves of serum total bilirubin and platelet resulted in an AUC of 0.69 (95% CI: 0.557–0.824, *p*=0.014) and 0.288 (95% CI: 0.128–0.449, *p*=0.006), respectively, using a cutoff value of 8.55umol/L for serum total bilirubin with a sensitivity of 93.8% and specificity of 40.9%, and a cutoff value of 28 (^*∗*^10^9^/L) for platelet with a sensitivity and specificity of 100% and 0%.

ROC curves resulted in an AUC of 0.69 for serum total bilirubin (95% CI: 0.557–0.824, *p*=0.014) and 0.288 for platelet (95% CI: 0.128–0.449, *p*=0.006), respectively. The cutoff value for serum total bilirubin was 8.55 *μ*mol/L with a sensitivity of 93.8% and specificity of 40.9%. For the platelet, the cutoff value was 28 × 10^9^/L with a sensitivity and specificity of 100% and 0%, respectively (Figure 4).

### 3.8. Cutoff Value of CRP and PCT after Treatment

**Figure 5:**
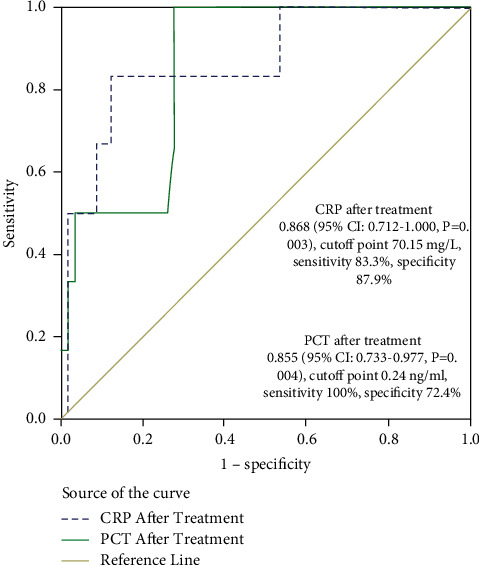
In order to identify the risk for death, we calculated the AUC of CRP and PCT after treatment which were 0.868 (95% Cl: 0.712–1.000, *p*=0.003) and 0.855 (95% Cl: 0.733–0.977, *p*=0.004) with cutoff values being 70.15 mg/L and 0.24 ng/ml, respectively. The sensitivity/specificity was 83.3%/87.9% and 100%/72.4%, respectively.

To identify the risk factors for mortality, we calculated the AUC of CRP and PCT after treatment, which yielded an AUC of 0.868 (95% CI: 0.712–1.000, *p*=0.003) and 0.855 (95% CI: 0.733–0.977, *p*=0.004), respectively. The cutoff values were 70.15 mg/L and 0.24 ng/ml. The sensitivity and specificity for CRP was 83.3% and 87.9%, while that for PCT was 100% and 72.4%, respectively (Figure 5).

## 4. Discussion

A large number of patients with severe pneumonia present hypoxic respiratory failure, which results in a high morbidity and mortality. In the past decades, extensive efforts have been made on the diagnosis and treatment of pneumonia with an aim to improve the outcome. In this study, we examined the feasibility of RAGE and OSM in predicting the outcome of pneumonia. In this retrospective study, our data showed that pneumonia with a lower oxygenation index was associated with gender, DM, APACHE II score, LIS, SOFA, NUTRIC score, PH value, lactic acid, WBC, neutrophils, lymphocyte, neutrophils/lymphocyte count, creatinine, D-dimer, PCT, CRP, RAGE, and albumin levels. Among these factors, the APACHE II score, LIS, SOFA, NUTRIC scores, WBC, neutrophils, lymphocyte count, RAGE and albumin levels were independent risk factors for severe pneumonia.

RAGE is constitutively highly expressed in type 1 and type 2 alveolar epithelial cells and vascular smooth muscle cells in lung [11]. It has been well accepted that RAGE is defined as a specific marker of ARDS [7]. For instance, RAGE can be targeted as new therapeutic strategies for the management of ARDS patients. [12] Besides, RAGE was closely associated with the pathogenesis of hypoxic pneumonia [13]. In our study, the increased serum RAGE level was an independent risk factor for hypoxemia in pneumonia patients. Our data suggested that RAGE might serve as a new biomarker for predicting hypoxemia of pneumonia even before the onset of ARDS.

Patients with severe pneumonia show a high morbidity and mortality. On this basis, screening of patients with high risks of severe pneumonia contributes to the early diagnosis and/or intervention, as well as delay in disease progression and even death. Some studies reported that lactate and lymphocyte could predict mortality in injured patients after resuscitation [14, 15]. Our data showed that LIS, SOFA, NUTRIC scores, lactic acid, lymphocyte, platelet, erythrocyte, BUN, LDH, total bilirubin, PCT, and OSM levels were significantly different between two groups. Meanwhile, LIS, SOFA scores, lactate, lymphocyte, platelet, BUN, total bilirubin, PCT, and OSM levels were proved to be independent predictive factors for a high mortality before treatment.

BUN was reported to be independently associated with mortality in critically ill patients [16]. Our data showed that the cutoff value of BUN was 9.22 mmol/L, which showed a sensitivity and specificity of 81.3% and 64.3% for the prediction of mortality. As previously described, elevation of serum bilirubin was associated with ARDS development and mortality in sepsis [17]. The cutoff value of serum bilirubin was 8.55 *μ*mol/L in our study, while the sensitivity and specificity of possibility for death was 93.8% and 40.9%, respectively. Moreover, post-treatment PCT and CRP, rather than pre-treatment PCT and CRP, were independent risk factors for 30-day mortality. Single factor logistic regression analysis revealed a cutoff value of 0.24 ng/ml for PCT, with a sensitivity of 100% and a specificity of 72.4%, respectively. In addition, the cutoff value for CRP was 70.15 mg/L, with a sensitivity of 83.3% and a specificity of 87.9%. These indicated that post-treatment PCT and CRP were risk factors for death. To date, there are still disputes on the roles of PCT in predicting the outcome of pneumonia. For example, some studies indicated that PCT was not an independent predictor of 30-day mortality in elderly and younger patients [18]. In contrast, some studies indicated that elevated PCT had moderate accuracy to identify poor outcome in septic patients [19]. However, we were among the very few groups that studied the relationship of PCT with disease morbidity after treatment.

In this study, we did not test the level of interleukin directly; however, the previous studies have shown that interleukin is closely related to the severity of pneumonia. For example, Han et al. showed that patients with COVID-19 pneumonia have higher serum level of cytokines (TNF-*α*, IFN- *γ*, IL-2, IL-4, IL-6 and IL-10) and CRP than control individuals. Serum IL-6 and IL-10 levels are significantly higher in the critical group than in the moderate and severe groups [20]. Also, Diane et al. found that high serum IL-6, IL-8, and TNF-*α* levels were strong and independent predictors of patient survival with COVID-19 pneumonia at the time of hospitalization [21]. As a member of the IL-6 family cytokines, OSM is closely involved in response to bacterial stimuli. However, little is known about its role in pneumonia. In this study, increased serum OSM at admission was associated with elevation of 30-day mortality. In a previous study, OSM may function as an important signal to epithelial cells for chemokines induction mediating neutrophil recruitment, which finally triggered the pathogenesis of severe inflammation [22]. Besides, it was also associated with increased vessel permeability as it could lead to endothelial barrier damages. Furthermore, OSM can also contribute to the activation of fibroblasts as well as multiple organ dysfunction during severe infection [23]. Consistently, our study showed that OSM was associated with prognosis of pneumonia. In future, more studies are needed to further confirm the predictive value of OSM in clinical practice.

It has been found that RAGE is related to OSM. Arunachalam et al. proved that there were enhanced plasma levels of inflammatory mediators, including EN-RAGE, TNFSF 14, and oncostatin-M (OSM) in severe COVID-19 infected patients, which was correlated with disease severity and increased bacterial products [24]. Moreover, knockout of receptor for advanced RAGE significantly attenuated cigarette smoke-induced airway inflammation in mice, and functional enrichment analyses showed the 14 functional methylated genes were enriched in immune-inflammatory responses, especially interleukin IL-6 and IL-17 pathways. This study suggested that RAGE mediated functional DNA methylated modification in a cluster of 14 targeted genes, particularly hypomethylation in promoter of OSM [25]. Therefore, we will focus on the correlation between OSM and RAGE in severe pneumonia.

There are really some limitations in our study. First, this was a retrospective study, which was inferior to the randomized controlled cohort study in strength. Second, the sample size was relatively small. Third, some parameters (e.g. blood pressure) that might affect oxygenation index were not included in this study.

## 5. Conclusions

In summary, APACHE II score, LIS, SOFA, NUTRIC score, WBC, neutrophils, lymphocyte counts, RAGE, and albumin levels were independent risk factors for severe pneumonia complicated with hypoxemia. RAGE showed a predictive value for severe injury in pneumonia. LIS, SOFA, lactate, lymphocyte, platelet, BUN, total bilirubin, PCT, and OSM levels were independent factors for 30-day mortality. In addition, OSM level upon RICU admission was associated with 30-day mortality.

## Figures and Tables

**Table 1:** Gender and comorbidities.

Variable	Oxygenation index >250 mmHg (*n* = 54)	Oxygenation index ≤250 mmHg (*n* = 76)	*Z* value	*p* value
Female	21	16	−2.212	0.027
Male	33	60		
Cerebrovascular disease	11	26	−1.717	0.086
Diabetes mellitus	7	21	−1.997	0.046
Coronary heart disease	19	31	−0.645	0.519
Chronic renal damage	4	12	−1.428	0.153
Treated nasopharyngeal carcinoma	0	2	−1.197	0.231

**Table 2:** Age, APACHE II, LIPS, SOFA, and NUTRIC scores.

Variable	Oxygenation index >250 mmHg (*n* = 54)	Oxygenation index ≤250 mmHg (*n* = 76)	*Z* value	*p* value
Age	69.63 ± 16.17	72.78 ± 14.94	−1.144	0.255
APACHE II	16.97 ± 6.12 (32)	20.03 ± 6.84 (72)	−2.17	0.032
LIS	3.13 ± 2.24 (32)	7.04 ± 2.78 (74)	−7.032	0.0001
SOFA	2.67 ± 2.60 (30)	6.34 ± 3.41 (74)	−5.294	0.0001
NUTRIC score	2.60 ± 2.71 (53)	5.46 ± 1.89 (76)	−7.069	0.0001

**Table 3:** Laboratory examination results at RICU admission.

Variable	Oxygenation index >250 mmHg (*n* = 54)	Oxygenation index ≤250 mmHg (*n* = 76)	*Z* value	*p* value
*Immunophenotyping*				
CD4	462.79 ± 171.08 (28)	421.08 ± 123.61 (65)	1.324	0.189
CD8	259.79 ± 98.31 (28)	259.49 ± 107.88 (65)	0.012	0.990
CD3	809.93 ± 239.67 (28)	764.83 ± 201.67 (64)	0.931	0.354

*Arterial blood gas*				
pH	7.44 ± 0.45(50)	7.41 ± 0.09 (76)	1.850	0.067
PaCO2 (mmHg)	38.56 ± 7.05(50)	41.66 ± 17.25 (76)	−1.206	0.230
Lac	1.47 ± 0.80(50)	1.88 ± 1.39 (76)	−1.880	0.063

*Blood cell analysis*				
WBC (109/L)	8.09 ± 4.16 (53)	10.71 ± 6.10 (76)	−2.717	0.008
Neutrophils (109/L)	6.09 ± 3.83 (53)	9.31 ± 5.71 (76)	−3.581	≤0.001
Lymphocyte (10^9^/L)	1.28 ± 0.80 (53)	0.86 ± 0.53 (76)	3.555	0.001
Hematocrit (%)	0.37 ± 0.07 (53)	0.37 ± 0.08 (76)	−0.277	0.782
Hemoglobin (g/L)	122.60 ± 21.85 (53)	121.04 ± 27.05 (76)	0.349	0.728
Platelet (10^9^/L)	199.74 ± 74.08 (53)	199.16 ± 105.09 (76)	0.034	0.973
Erythrocyte (10^12^/L)	4.07 ± 0.78 (53)	4.10 ± 0.94 (76)	−0.142	0.888
Neutrophils/Lymphocyte (%)	9.32 ± 16.40 (51)	15.13 ± 15.98 (76)	−1.989	0.049

*Biochemical analysis*				
Albumin (g/L)	35.80 ± 7.70 (51)	30.38 ± 4.53 (75)	4.962	≤0.001
BUN (mmol/L)	6.79 ± 5.08 (52)	19.66 ± 59.82 (76)	−1.545	0.125
Creatinine (µmol/L)	87.43 ± 96.15 (52)	123.60 ± 102.80 (76)	−2.007	0.047
LDH (IU/L)	375.90 ± 178.88 (51)	542.54 ± 716.54 (76)	−1.625	0.107
Total bilirubin (µmol/L)	12.17 ± 5.63 (51)	12.45 ± 10.06 (75)	−0.184	0.854

*Coagulation analysis*				
PT (s)	12.61 ± 4.10 (52)	12.85 ± 2.48 (76)	−0.405	0.686
APTT(s)	29.97 ± 5.91(52)	30.67 ± 7.01(76**)**	−0.587	0.558
FiB (g/L)	4.54 ± 1.60 (52)	5.35 ± 4.69 (76)	−1.194	0.235
D-dimer	1.31 ± 1.91(52)	5.66 ± 8.68 (76)	−3.549	0.001

*Inflammatory biomarkers*				
Procalcitonin (ng/mL)	0.61 ± 1.60 (48)	6.98 ± 20.91 (71)	−2.105	0.037
CRP (ng/L)	59.83 ± 64.33 (51)	113.59 ± 96.67 (76)	−3.487	0.001
OSM (pg/mg)	54.85 ± 58.64 (53)	80.32 ± 110.43 (74)	−1.530	0.128
RAGE (pg/ml)	632.62 ± 469.73 (53)	1034.26 ± 1068.24	−2.563	0.012

**Table 4:** Gender and comorbidities between survival and nonsurvival patients.

Variable	Non-survival group (*n* = 16)	Survival group (*n* = 114)	*Z* value	*p* value
Female	3	34	−0.916	0.360
Male	16	80		
Cerebrovascular disease	1	36	−2.095	0.086
Diabetes mellitus	5	23	−1.005	0.315
Coronary heart disease	9	41	−1.556	0.120
Chronic renal damage	3	13	−0.834	0.404
Treated nasopharyngeal carcinoma	0	2	−0.532	0.595

**Table 5:** Age, APACHE II, LIPS, SOFA and NUTRIC scores between survival and nonsurvival patients.

Variable	Non-survival group (*n* = 16)	Survival group (*n* = 114)	*Z* value	*p* value
Age	73.44 ± 12.36	71.19 ± 15.89	0.542	0.589
APACHE II	20.93 ± 6.02 (15)	18.77 ± 6.84 (89)	1.148	0.254
LIS	7.56 ± 3.93(16)	5.56 ± 2.95 (90)	2.376	0.019
SOFA	7.69 ± 5.04(16)	4.84 ± 3.11 (113)	3.022	0.003
NUTRIC score	7.69 ± 5.04(16)	4.09 ± 2.68 (113)	2.292	0.024

**Table 6:** Laboratory examination results between survival and nonsurvival patients before treatment.

Variable	Non-survival group (*n* = 16)	Survival group (*n* = 114)	*Z* value	*p* value
*Immunophenotyping*				
CD4	381.33 ± 35.13 (12)	441.38 ± 147.94 (81)	−1.394	0.167
CD8	221.50 ± 26.78 (12)	265.22 ± 110.56 (81)	−1.358	0.178
CD3	681.33 ± 77.79 (12)	793.14 ± 223.77 (80)	−1.708	0.091

*Arterial blood gas*				
pH	7.44 ± 0.08 (16)	7.42 ± 0.08 (110)	1.074	0.285
PaCO2 (mmHg)	39.59 ± 8.41(16)	40.55 ± 14.83 (110)	−0.252	0.801
Lac	2.46 ± 2.13 (16)	1.61 ± 0.98 (110)	2.676	0.008

*Blood cell analysis*				
WBC (10^9^/L)	8.30 ± 4.63 (16)	9.83 ± 5.63 (113)	−1.034	0.303
Neutrophils (10^9^/L)	7.26 ± 4.55 (16)	8.09 ± 5.36 (113)	−0.587	0.558
Lymphocyte (10^9^/L)	0.73 ± 0.59 (16)	1.078 ± 0.69 (113)	−1.941	0.054
Hematocrit (%)	0.35 ± 0.08 (16)	0.37 ± 0.08 (113)	−0.852	0.396
Hemoglobin (g/L)	115.56 ± 25.18 (16)	122.55 ± 24.93 (113)	−1.048	0.297
Platelet (109/L)	139.88 ± 81.63 (16)	207.82 ± 92.05 (113)	−2.799	0.006
Erythrocyte (10^12^/L)	3.74 ± 0.72 (16)	4.13 ± 0.89 (113)	−1.691	0.093
Neutrophils/Lymphocyte (%)	13.94 ± 12.15 (16)	12.63 ± 16.89 (111)	0.298	0.766

*Biochemical analysis*				
Albumin (g/L)	33.59 ± 11.41(16)	32.43 ± 5.59 (110)	0.664	0.508
BUN (mmol/L)	48.27 ± 128.46 (16)	9.60 ± 7.56 (112)	3.223	0.002
Creatinine (µmol/L)	113.06 ± 81.79 (16)	108.31 ± 104.13 (112)	0.175	0.862
LDH (IU/L)	710.81 ± 948.51(16)	441.72 ± 490.33 (111)	1.780	0.071
Total bilirubin (µmol/L)	17.49 ± 12.15 (16)	11.59 ± 7.65 (110)	2.651	0.009

*Coagulation analysis*				
PT (s)	13.23 ± 3.25 (16)	12.68 ± 3.23 (112)	0.626	0.533
APTT(s)	31.43 ± 9.16 (16)	30.24 ± 6.15 (112)	0.675	0.501
FiB (g/L)	3.85 ± 2.23 (16)	5.19 ± 3.91 (112)	−1.328	0.187
D-dimer	6.12 ± 8.74 (16)	3.58 ± 6.83 (112)	1.342	0.182

*Inflammatory biomarkers*				
Procalcitonin (ng/mL)	14.68 ± 36.15 (14)	3.04 ± 11.28 (105)	2.545	0.012
CRP (ng/L)	88.24 ± 107.42 (16)	92.54 ± 86.45 (111)	−0.180	0.857
OSM (pg/mg)	142.63 ± 196.72 (15)	59.92 ± 64.15 (112)	3.366	0.001
RAGE (pg/ml)	1099.89 ± 810.56 (15)	835.41 ± 898.62 (112)	1.082	0.281

**Table 7:** Laboratory examination results before and after one-week treatment in the survival group.

Variable	Survival	*T* value	*p* value
Lac	−0.77 ± 9.64	−0.692	0.491
PH	−0.03 ± 0.08	−3.184	0.002
PaCO_2_	−1.81 ± 13.27	−1.173	0.245
WBC	1.11 ± 6.24	1.536	0.129
Neutrophils	1.52 ± 6.00	2.182	0.032
Lymphocyte	−0.22 ± 0.82	−2.340	0.022
RBC	0.41 ± 0.56	6.271	≤0.001
Hemoglobin	17.79 ± 17.26	6.602	≤0.001
Hematocrit	0.04 ± 0.05	5.761	≤0.001
Platelet	−66.54 ± 100.87	−4.224	≤0.001
Albumin	−3.70 ± 5.45	−4.344	≤0.001
Creatinine	23.79 ± 53.28	2.859	0.007
BUN	−0.26 ± 6.15	−0.274	0.785
Total bilirubin	3.39 ± 9.91	2.187	0.035
PT	0.30 ± 3.57	0.533	0.597
APTT	0.47 ± 6.89	0.440	0.663
FiB	1.62 ± 6.16	1.685	0.100
CRP	92.20 ± 77.36	7.632	≤0.001
PCT	5.02 ± 12.87	2.501	0.017
OSM	43.64 ± 68.11	5.086	≤0.001
RAGE	379.09 ± 891.45	3.375	0.001
Neutrophils/Lymphocyte	6.71 ± 14.86	3.587	0.001

**Table 8:** Laboratory examination results before and after one-week treatment in the nonsurvival group.

Variable	Survival	*T* value	*p* value
Lac	−2.41 ± 4.31	−1.853	0.094
PH	−0.150 ± 0.13	3.878	0.003
PaCO_2_	−16.20 ± 19.20	−2.799	0.019
WBC	−1.85 ± 4.36	−1.409	0.189
Neutrophils	−2.21 ± 4.11	−1.409	0.189
Lymphocyte	0.38 ± 0.79	1.579	0.145
RBC	0.40 ± 0.66	2.020	0.071
Hemoglobin	18.40 ± 22.14	1.858	0.137
Hematocrit	0.05 ± 0.06	1.783	0.149
Platelet	−36.20 ± 86.04	−0.941	0.400
Albumin	3.92 ± 5.87	1.492	0.210
Creatinine	−68.80 ± 94.59	−1.626	0.179
BUN	92.38 ± 234.38	0.867	0.435
Total bilirubin	−14.22 ± 26.91	−1.182	0.303
PT	−2.08 ± 1.97	−2.359	0.078
APTT	−4.52 ± 5.79	−1.746	0.156
FiB	−2.42 ± 2.66	−2.032	0.112
CRP	−73.86 ± 48.48	−3.406	0.027
PCT	−9.74 ± 21.28	−1.023	0.364
OSM	−14.73 ± 50.73	−0.711	0.509
RAGE	163.81 ± 1544.84	0.260	0.805
Neutrophils/Lymphocyte	−10.36 ± 19.58	−1.296	0.252

## Data Availability

The data used to support the findings of this study are available from the corresponding author upon request.

## Abbreviations

RICU: Respiratory intensive care unit
APACHE II: Acute physiology, age, chronic health evaluation
LIS: Lung injury score; SOFA: sequential organ failure assessment
NUTRIC score: The nutrition risk in critically ill score
PCT: Procalcitonin
CRP: C-reactive protein
ARDS: Acute respiratory distress syndrome
CD4: CD4+T cell
CD8: CD8+T cell
CD3: CD3+T cell
PH: Potential of hydrogen
PaCO2: Partial pressure of arterial carbon dioxide
Lac: Lactic acid
WBC: White blood cell count
BUN: Blood urea nitrogen
LDH: Lactate dehydrogenase
PT: Prothrombin time
APTT: Activated partial thromboplastin time
FiB: Fibrinogen
RAGE: The receptor for advanced glycation end products
OSM: Oncostatin M
AUC: Area under the curve
CI: Confidence interval
ROC: Receiver operating characteristic.

## Supplementary Materials

Supplementary Materials The supplementary materials include the following: Word file: STROBE_checklist_v4_combined_PlosMedicine; PDF file: Jiangnan Zhao's consent; PDF file: Yi Shi's consent.
